# Analgesic efficacy of ultrasound guided unilateral erector spinae plane block for laparoscopic inguinal hernia repair: a randomized controlled study

**DOI:** 10.55730/1300-0144.5355

**Published:** 2022-01-01

**Authors:** Savaş ALTINSOY, Derya ÖZKAN, Fatma KAVAK AKELMA, Jülide ERGİL

**Affiliations:** Department of Anaesthesiology and Reanimation, Dışkapı Yıldırım Beyazıt Training and Research Hospital, University of Health Sciences, Ankara, Turkey

**Keywords:** Erector spinae plane block, acute postoperative pain, inguinal hernia, postsurgical recovery

## Abstract

**Background/aim:**

Although laparoscopic inguinal herniorrhaphy is associated with faster return to daily activity, inadequate postoperative pain control can cause prolonged hospital stays and patient discomfort. Erector spinae plane block (ESP) can be administered for postoperative pain management in abdominal and thoracic surgery. We investigated the effects of unilateral ESP block application in laparoscopic hernia surgery.

**Materials and methods:**

A total of 60 patients who were scheduled for laparoscopic inguinal hernia surgery were included. ESP block was performed in group E (n = 30) after induction of anesthesia. There was no intervention in Group C (n = 30). Postoperative pain was assessed by the patient using the numeric rating scale (NRS) at postanesthetic care unit (PACU),1, 4, 6, 12, and 24 h after surgery. The quality of postoperative functional recovery was evaluated using the quality of recovery-40 questionnaire.

**Results:**

NRS scores were lower in Group E (n = 30) than in Group C (n = 30) at PACU, 1^th^, 4^th^, 6^th^ hours in both rest and movement. Total tramadol consumption was reduced at postoperative 24 h by the ESP block [median(IQR), 60(40) versus 85(30)]. Quality of recovery score of the patients after operation was better in the ESP group than in the control group [mean(SD), 177.9(6.5) in group E and 173.2(7.09) in group C with mean differences: 4.633 and CI: 95% (1.11 to 8.15) respectively].

**Conclusions:**

Unilateral ESP block s in laparoscopic inguinal hernia surgery reduce both postoperative pain levels and analgesic consumption. In addition, the ESP block could be used safely in pain management of this type of surgery and improve the quality of recovery.

## 1. Introduction

Inguinal hernia is a common condition seen in nearly 3.8% (20 million) of the population and due to its high incidence, this condition is frequently observed in general surgical practice [1]. Although laparoscopic inguinal herniorrhaphy is associated with shorter hospital stays and return to normal daily activity quicker than seen with open surgery, inadequate postoperative pain control can cause prolonged hospital stays and patient discomfort [2–4].

The pain observed in laparoscopic inguinal herniorrhaphy usually occurs on the first postoperative day and usually increases with coughing and movement [5]. Recently, regional blocks (transversus abdominis plane [TAP] and rectus sheath blocks), intravenous [iv] lidocaine were investigated as a part of a multimodal approach for postoperative pain management in inguinal herniorrhaphy [5,6]. In fact, in procedure-specific pain management (PROSPECT) recommendations, multimodal approaches including peripheral nerve blocks are found [7]. New block techniques, such as erector spinae plane (ESP), have not yet been included in the current recommendations.

In the ESP block, the local anesthetic administered at the different levels of the transverse process spreads in the related paravertebral spaces, leading to effective analgesia for somatic and visceral pain [8]. However, information on the effectiveness of the ESP block in laparoscopic inguinal herniorrhaphy is limited.

The quality of recovery 40 (QoR-40) questionnaire, which includes increasing comfort and rapid resumption of normal activities, has been used in outpatient surgery to assess the difference in recovery quality depending on the type of anesthesia or analgesia [9].

The primary aim of this study was to evaluate whether the unilateral ESP block would cause a decrease in pain during periods of rest and movement compared to a control group in the first 24 h after laparoscopic herniorrhaphy. Postoperative nausea and vomiting, complications like pneumothorax, local anesthetic toxicity, and bleeding related to the nerve block, peri-operative opioid consumption and the quality of recovery (QoR) of the patients were also evaluated.

## 2. Materials and methods

**Figure 1:**
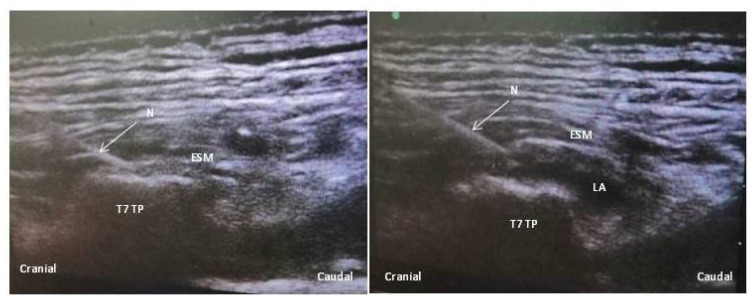
Local anesthetic spread and sonoatanomy (TP: Transverse process, ESM: Erector Spinae Muscle, N: Needle, LA: Local anesthetic)

Ethical approval for this study (Ref no 66/01) was provided by the Ethical Committee of the Ministry of Health, D**ışkapı** Y**ı**ldır**ı**m Beyaz**ı**t Training and Research Hospital, Ankara, Turkey on June 28, 2019. The study was registered in clinicaltrials.gov (NCT04131985). Written informed consent was obtained from all the patients. Sixty adult patients, aged 18–70 years and classified as American Society of Anaesthesiologists (ASA) status I–III, who were scheduled for laparoscopic inguinal herniorrhaphy between October 2019 and January 2020 were included (Figure 1). Patients with history of decompensated cardiorespiratory diseases, renal disorders, acute pancreatitis, cognitive disorders (inability to comprehend or use the numeric rating pain scoring system or a patient-controlled analgesia [PCA] pump), allergies to any study drugs, had skin infections at the site of needle puncture area; and from whom written informed consent could not be obtained were excluded. On the other hand, patients who were transitioned to open surgical intervention were also excluded.

Preoperative visits were conducted for all of the patients by an anaesthesiologist, and the patients were instructed in the use of the numeric rating scale (NRS) for pain assessment (0 = no pain; 10 = worst pain imaginable) and a system for patient-controlled analgesia (PCA). After anesthesia induction, patients were randomly allocated into two groups Group E (n = 30) and Group C (n = 30) (1:1 allocation ratio) according to a randomized sequence by using a computer-generated list; these sequences were kept in sealed and consecutively numbered opaque envelopes, which were opened at the day of surgery. All the patients received midazolam (0.05 mg/kg) administered i.v. as premedication 30 min before surgery. Peripheral venous catheterizations of patients in both groups were performed with 20 gauge needles and Ringer lactate (2 ml/kg) was administered before operation during the fasting period. Following routine monitoring with electrocardiography (ECG), pulse oximetry, end-tidal carbon dioxide (EtCO_2_) and BIS monitorization (BIS Quatro sensor and BIS VISTA monitor), anaesthesia was induced with fentanyl citrate (1 μg/kg), propofol (2 mg/kg). All patients received rocuronium 0.6 mg/kg as neuromuscular blockade before intubation. Anesthesia was maintained with 80–100 μg/kg/min propofol and remifentanil infusion at a dose of 0.05–0.1 μg/kg/min. The doses of propofol and remifentanil were adjusted to achieve BIS values between 40–60 and hemodynamic response (30% changes in hemodynamic values). Granisetron (40 μg/kg) was performed for postoperative nausea and vomiting (PONV) prophylaxis to all patients just before the induction of anesthesia.

In Group E, ESP block was performed hernia side, unilaterally by an experienced anesthesiologist (DO) who was blinded to the data collection in the lateral position at T7 vertebral level after intubation [10]. A low-frequency convex probe was placed 2–3 cm lateral to the spine using a sagittal approach and erector spinae muscle was identified above the transverse processes. The needle was inserted in a cranio-caudal direction to deep to the erector spinae muscle. The correct position of the needle tip was controlled with the administration of 0.5–1 mL of LA. Afterward, 20 mL of 0.25% bupivacaine was administered for the block. The LA distribution was seen as both cranial and caudal directions (Figure 1). Although there was no intervention in control group, all patients were covered at T7 vertebral level with a sterile cover as Group E for blinding.

Analgesic management was maintained using 50 mg of dexketoprofen trometamol following anesthesia induction. IV tramadol (100 mg) was administered to the patients in two groups approximately 15 min before the end of the surgery. Upon discharge from the postanesthesia care unit (PACU), all patients received iv tramadol infusion via PCA (20 mg bolus with a 20-min lockout period) device (CADD-Legacy^®^ PCA pump, Smiths Medical, USA) for 24 h. Postoperative pain was assessed and recorded by the patient using the numerical rating scale (NRS) in the PACU (when modified Aldrete score was >9) at 1, 4, 6, 12, and 24 h after surgery. If the patients complained of pain (NRS ^3^ 4), dexketoprofen trometamol 50 mg, iv was administered. Total tramadol consumption during the first 1, 4, 6, 12, and 24 h was recorded. Postoperative nausea and vomiting (PONV) and complications related to the nerve block (pneumothorax, local anesthetic toxicity, and bleeding) were recorded.

In both groups, the sensory block levels were measured by using a 4-point scale (0 = no cold, 3 = normal cold) with a bilateral cold stimulation test in both dermatomes between the mid-axillary and mid-abdominal line in the PACU.

The quality of functional postoperative recovery was evaluated using the QoR-40 questionnaire, which evaluates physical comfort (12 items), emotional state (9 items), physical independence (5 items), psychological support (7 items), and pain (seven items). Each item was rated on a 5-point Likert scale ranging from 1 (none of the time) to 5 (all the time). The total score ranged from 40 (poorest quality of recovery) to 200 (the best quality of recovery). The validity and reliability parameters of the QoR-40 score in the Turkish population were established by Karaman et al. [11]. The QoR-40 was administered one day before surgery in outpatient clinics of anesthesiology (t1), at 6 h after the surgery (t2), and before discharge from the hospital on the first postoperative day (t3) [12].

Peri-operatively, all assessments were recorded by a researcher who was blinded to group allocation.

### 2.1. Statistical analysis

We performed a priori sample size calculation, based on pilot data obtained from postoperative NRS scores, using a difference between two independent means (two groups) test with the G*Power version 3.1.9.2 (© Franz Faul, Edgar Erdfelder, Albert-Georg Lang, and Axel Buchner, 2006, 2009). In the preliminary study, the NRS score (mean ± SD) was 2.9 ± 1.03 in the control group. According to the studies in the literature, it has been shown that the difference of 1 unit in NRS values in inguinal herniorrhaphy is clinically significant [13,14]. Accordingly, using α-error = 0.05 at 95% power, the number of patients required per group was determined as 29. Considering potential dropouts, we included 35 patients in each group.

NRS score differences between groups were assessed by fitting a cumulative link mixed model (CLMM) with NRS scores as the dependent variable, patient as random effect and group, and the opioid dose in 24 h and interaction between the study group and postoperative hour as independent variables. The common within-patient association for paired measurements of Tramadol and NRS at rest/movement on different time points was assessed with repeated measures correlation proposed by Bakdash and Marusich [15]. R programming language was used for data analysis. Cumulative link mixed models were fitted with “*clmm2”* function in ***ordinal*** package, whereas repeated measures correlations along with their 95% confidence intervals were computed with “*rmcorr”* function in ***rmcorr*** package. The Statistical Package for the Social Sciences (SPSS, version 22.0, Chicago, IL, USA) was used for analysis. Numerical values are expressed as the mean ± standard deviation and median (min–max). The Shapiro-Wilk test was used to check normality. The student’s test-test was used for continuous variables with a normal distribution. The Mann-Whitney U tests were used for continuous variables without normal distribution. Chi-square tests were used for categorical variables. For multiple comparisons variance analyses and paired test-test was used. P < 0.05 was considered statistically significant.

## 3. Results

**Figure 2:**
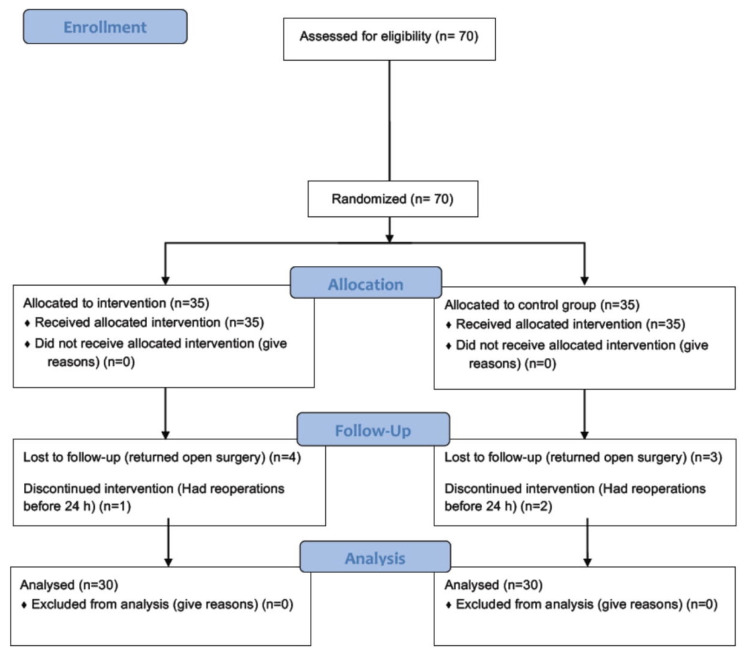
Flow diagram of study.

Seventy patients were enrolled in the study. Four patients in Group E and three in Group C were returned to open surgery and excluded from the study. One patient in Group E and two in Group C had reoperations before 24 h and were excluded from the study. Sixty patients were ultimately analyzed (Figure 2).

The demographic variables were similar in both groups. Duration of anesthesia (time from induction until transfer to the PACU) and surgery (time between the first incision and last dressing applied) are demonstrated in Table 1. Intraoperative propofol consumption was similar in both groups [mean(SD) 428(53.2) and 431(52.4) respectively] [with mean differences: 3.06 and 95%CI: (−24.24 to −30.37)] (p > 0.05). Intraoperative remifentanil consumption was lower in Group E than in Group C [ mean(SD), 494(100.02) and 624(105.18) respectively] [mean differences: −130.83 and 95%CI: (−183.88 to −77.78)] (p < 0.001).

The sensory block was observed in 25 patients on bilateral dermatomes in the ESP group. On the block-treated side, the upper limit of sensory block level was T5 in 20 patients, T6 in 4 patients, and T7 in 6 patients, and the caudal limit of sensory block level was T12 in 22 patients and L1 in 8 patients. On the contralateral side, the upper limit of sensory block level was T7 in 25 patients, and the caudal limit of sensory block was T10 in 9 patients and T12 in 16 patients.

**Figure 3:**
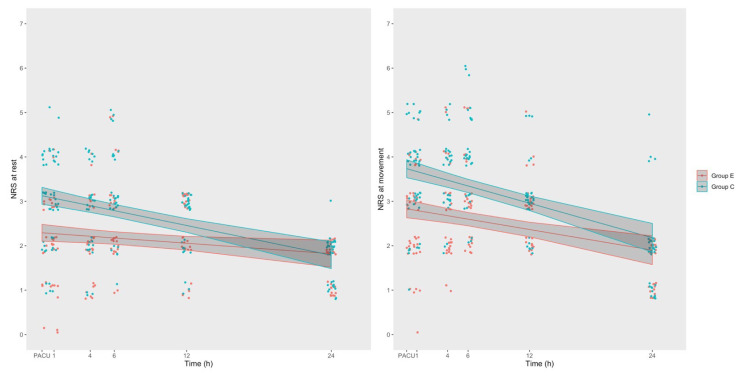
Cumulative link mixed regression model. Numeric rating scale (NRS) pain evolution during follow-up periods. Solid line: median tendency. Fade color area: 95% confidence area. Jitter was added to each value’s points to improve visibility. (E; group E, C; group C, PACU; postanaesthesia care unit)

The postoperative NRS scores at rest and coughing are presented in Table 2. Data, according to the cumulative link mixed model, are presented in Table 3. The probability of having NRS scores at rest and movement above a certain value (e.g., NRS ≥ 2) in Group C are, respectively, 5.250 (95% CI: 2.327–11.839) and 4.870 (95% CI: 2.267–10.458) times higher than in Group E. In other words, being in study Group C can be said to have a risk of higher NRS scores at rest. The odds ratio (OR) is adjusted for inter-patient variability (via random effect), opioid dose, and postop hour. The NRS scores of Group E patients were significantly lower through the 24 h follow-up period (Figure 3).

**Figure 4:**
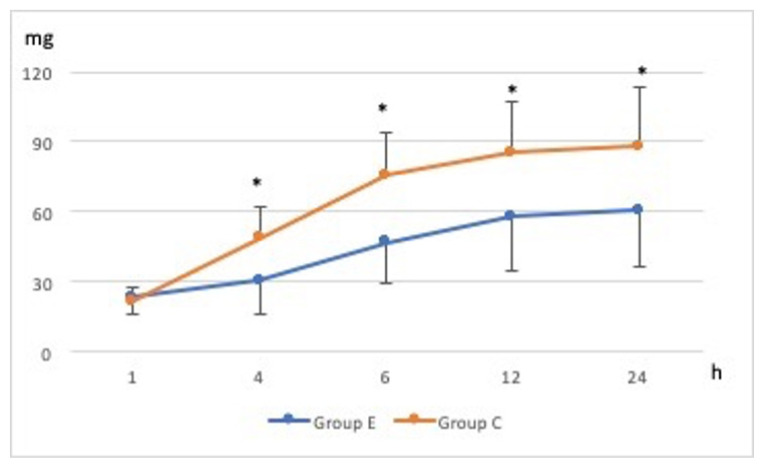
Cumulative tramadol consumption of patients during follow-up periods (*; p < 0.05).

Total tramadol consumption was significantly lower in Group E than in Group C when the groups were compared [mean(SD), 60.6(24.34) and 88.0(25.5) respectively] [mean difference: −27.33 and 95%CI: (−40.22 to −14.44)] (p < 0.001). Cumulative tramadol consumption during follow-up periods is demonstrated in Figure 4 and was lower in Group E than in Group C at 1, 4, 6, 12, and 24 h (p < 0.05).

**Figure 5:**
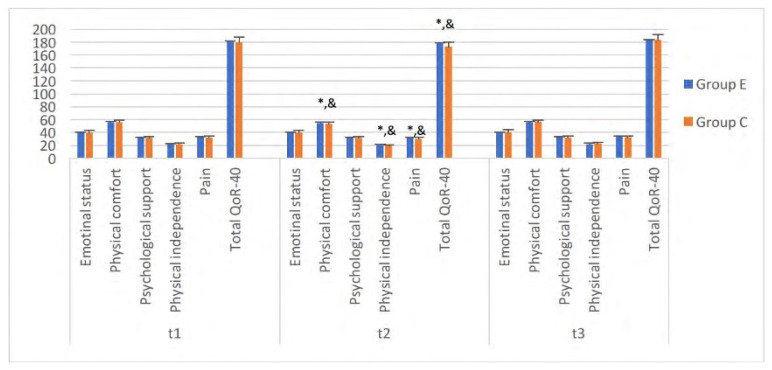
QoR-40 score of patients between groups (t1; one day before surgery, t2; 6 h after the surgery, t3; before discharged from hospital on first postoperative day, *; compared with t1, &: differences between groups).

QoR-40 scores of the groups were lower at 6th h compared to baseline values [mean(SD), 181(8) versus 177(6) in group E and 179(8) versus 173(7) in group C] (p<0.001). Comparing the groups, higher scores were observed in Group E than in Group C at 6 h (p < 0.05) (Figure 5). When the QoR-40 score subtypes were analyzed, it was seen that this difference was mainly with regard to pain [mean(SD), 31(2.5) versus 29(2.3), p = 0.016], physical comfort [mean(SD), 54(2.7) versus 53(2.8) p = 0.022], and physical independence [mean(SD), 20(1.5) versus 19(1.1),p = 0.005] .

Pneumothorax, local anesthetic toxicity, and bleeding were not observed in either group. The number of patients in Group E experiencing postoperative nausea and vomiting was less than in Group C (respectively n = 2 versus n = 8) (p = 0.038). There were no significant differences between the groups on rescue analgesic consumption (respectively 100 mg versus 150 mg).

## 4. Discussion

In this study, results showed that the unilateral ESP block provided good conditions for postoperative analgesia management in laparoscopic inguinal herniorrhaphy patients. NRS scores were lower in Group E than in Group C at least at 6 h postoperatively. Total tramadol consumption was reduced at 24 h postoperatively by the ESP block. QoR scores of the patients after operation were better in the ESP group than in the control group.

Laparoscopic procedures are preferred because they result in less postoperative pain, faster healing times, and better cosmetic results than open surgeries. Although laparoscopic inguinal herniorrhaphy is applied in two methods, total extraperitoneal (TEP) and transabdominal preperitoneal (TAPP), the pain associated with the two types is similar [2,5]. This pain tends to be moderate or severe, usually increasing with coughing and movement, especially on the first postoperative day. Although there is no complete consensus, it is known that sensory block at T4 dermatomal levels and below provides adequate anesthesia in abdominal surgery. Unlike open surgery pain after both TAPP and TEP repairs is deep, abdominal groin pain due to hernia repair, dominates over somatic or incisional pain, and is more pronounced than shoulder pain due to pneumoperitoneum [2,5]. Furthermore, no relationship was found between the size of the trocar and the severity of pain [16]. In the literature, ESP block was performed at the level of T7 for abdominal surgery and its sensorial effects were distributed between T6 and T12 dermatomes [8,10]. In a cadaver study, it is stated that 20 mL of contrast agent applied from T7 ESP level shows the wide craniocaudal spread between C5-T2 and L2-L3 levels [10]. The columnar arrangement of the erector spinae muscle and the associated retinaculum provides an anatomical basis for extensive cranio-caudal diffusion of fluid injected into the ESP, and also means that injection between the caudal to T5 levels must affect the nerve roots that supply the abdomen with lower thoracic extension. According to these data we preferred T7 level to administer the ESP block.

Unilateral ESP block application in laparoscopic herniorrhaphy may cause controversy since the placement of trocars includes the contralateral side of the hernia. In cadaveric and magnetic resonance imaging (MRI) studies performed with unilateral ESP block, it was observed that the contrast agent delivered through the superior costotransverse ligament reached the paravertebral area and thoracic spinal nerves [17–19]. Tulgar et al. also detected bilateral sensory block after ESP block unilaterally at the T9 level [20]. In this study, the sensory block was observed in 25 patients on bilateral dermatomes in the ESP group and sensorial block levels are distributed between T5 and L1 dermatomes on the block-treated side and between T7 and T12 on the contralateral side. This situation could be explained by the contralateral spread of local anesthetic through the epidural space, similar to reports in the literature [21,22]. NRS and QoR scores were acceptable in 5 patients without bilateral sensory block and in patients whose caudal sensory border remained at L1. This could be explained by the use of multimodal analgesia.

Many methods, such as TAP, port site infiltration of a local anesthetic or preperitoneal injection, iv lidocaine, and recently quadratus lumborum block, are used in pain management after laparoscopic surgical procedures [6,23–25]. However, some limitations to using of these techniques can be found. For example, conventional approaches, such as TAP block, provide effective somatic analgesia with minimal or no visceral blockade [26]. The need for the visceral blockade to provide better postoperative pain relief led to a more posterior approach that involves injecting the local anesthetic adjacent to the quadratus lumborum (QL) muscle [27,28]. Although USG guidance, some physical complications due to quadratus lumborum block (QLB) associated with a needle puncture, such as abdominal wall hematoma, vascular injury, bowel or diaphragm perforation, and liver damage are dubious but still present a risk [29]. With respect to iv lidocaine infusion, definite improvements in postoperative pain compared to placebo or no treatment at early time points (1–4 h) are not reported in the literature [30]. Local anesthetic toxicity is another problem for limiting the use of all plane blocks especially in cases of bilateral administration.

ESP block has an effect on both somatic fibers carrying pain caused by the skin and visceral fibers carrying pain signals due to peritoneal irritation. The ESP block has some advantages: first, it is simple with easily identifiable ultrasonographic landmarks and endpoint for injection, and second, low risk for serious complications because the injection is into a tissue plane that is distant from pleura, major blood vessels, and discrete nerves. Pneumothorax, local anesthetic toxicity, and bleeding were not observed in this study during the peri-operative period.

ESP block was reported in the literature in different types of procedures, and good results were obtained with respect to postoperative opioid consumption and pain scores compared to control groups [31]. In the current study, NRS levels and postoperative tramadol consumption were lower in Group E compared to Group C both during periods of movement and rest.

The most important advantage of regional techniques is to reduce the need for opioids in both the intra- and postoperative periods, to shorten the length of hospital stay, and to prevent opioid-related side effects. Wang et al. [32] performed ESP blocks for thoracotomy pain and emphasized that patients needed less intraoperative analgesics than the infiltration group. They also reported less nausea and vomiting in their ESP group and attributed this to decreased opioid consumption in the postoperative period. Similarly, in this study, remifentanil consumption in the intra-operative period and tramadol consumption in the postoperative period were lower in the ESP group. The number of patients in Group E experiencing postoperative nausea and vomiting was less than in Group C.

The relationship between pain and recovery is well known. The quality of postoperative functional recovery has been reported as more successful after applying peripheral blocks [33]. QoR-40 score is widely used in the evaluation of this period. In the current study, the QoR-40 score was applied at three-time points: (1) preoperative, (2) sixth postoperative h, and (3) before discharge from the hospital on the first postoperative day. Especially at h 6, high-quality scores were obtained in Group E. We think that this difference was due to decreased pain, nausea, vomiting, emotional comfort, and physical independence (breathing, moving) in the ESP block group. The ESP block may have resulted in a decrease in postoperative pain levels, less nausea and vomiting and an increase in the feeling of wellbeing due to lower opioid consumption.

The study had some limitations, first, patients were not followed up after 24 h. Second, the patients were compared with the nonintervention group. In future studies, it would be appropriate to evaluate patients in terms of chronic pain in the long term and to compare ESP blocks with other blocks.

In conclusion, unilateral ESP blocks in laparoscopic inguinal herniorrhaphy can reduce both postoperative pain levels and analgesic consumption. In addition, the ESP block could be used safely in pain management of this type of surgery and improve the QoR.

## Figures and Tables

**Table 1:** Demographic data and perioperative detail of the patients.

	Group E (n = 30)	Group C (n = 30)	p
Age (year)(mean ± SD)	61.3 ± 6.8	60.6 ± 6.1	0.680
Gender (n) Female/male	8/22	9/21	0.774
ASA (n) I/II/III	12/10/8	12/11/7	0.944
BMI (m^2^/kg)(mean ± SD)	28.1 ± 2.4	27.9 ± 2.7	0.766
Type of surgery (n) TEP/ TAPP	22/8	24/6	0.542
Duration of anesthesia (min)(mean ± SD)	81.03 ± 8.6	77.4 ± 7.5	0.089
Duration of surgery (min)(mean ± SD)	70.5 ± 7.4	71.3 ± 8.6	0.691

TEP: Total extraperitoneal, TAPP: Transabdominal preperitoneal, BMI: Body mass index, ASA: American Society of Anesthesiologists status

**Table 2:** NRS scores.

	Group E (n = 30)	Group C (n = 30)	p
**At rest**			
PACU	2 (2)	3 (2)	0.004 *
1 h	2 (1.25)	3 (2)	0.004 *
4 h	2 (1.25)	3 (1.25)	0.009 *
6 h	2.5 (1)	3 (1)	0.021 *
12 h	2.5 (1)	3 (1)	0.242
24 h	2 (1)	2 (1)	0.677
**At movement**			
PACU	2.5 (1)	3 (2)	0.015 *
1 h	2 (1)	4 (1)	0.000 *
4 h	3 (0.2)	4 (1)	0.002 *
6 h	3 (2)	4 (2)	0.001 *
12 h	3 (1)	3 (0.25)	0.284
24 h	2 (1)	2 (1)	0.185

* p < 0.05 is significant, data was presented as median(IQR), PACU: postanaesthesia care unit

**Table 3:** Cumulative link mixed model (CLMM) with NRS score as dependent variable.

	Estimate	SE	OR	Lower 95%CI	Upper 95%CI	p
NRS scores at rest						
Group E	1.658	0.415	5.250	2.327	11.839	<0.001
Hour	−0.046	0.017	0.955	0.924	0.987	0.008
Opioid dose	0.007	0.006	1.007	0.995	1.019	0.307
Group*Hour	−0.078	0.024	0.925	0.882	0.970	0.001
NRS scores at movement						
Group E	1.583	0.390	4.870	2.267	10.458	<0.001
Hour	−0.095	0.018	0.909	0.878	0.942	<0.001
Opioid dose	0.006	0.006	1.006	0.994	1.018	0.325
Group*Hour	−0.047	0.024	0.954	0.910	1.00	0.052

Estimate: CLMM regression coefficient, SE: Standard error of regression coefficients, OR: Odds ratio, 95%CI: 95% Confidence interval
